# Response to *M. tuberculosis *selected RD1 peptides in Ugandan HIV-infected patients with smear positive pulmonary tuberculosis: a pilot study

**DOI:** 10.1186/1471-2334-8-11

**Published:** 2008-01-28

**Authors:** Delia Goletti, Stefania Carrara, Harriet Mayanja-Kizza, Joy Baseke, Michael Angel Mugerwa, Enrico Girardi, Zahra Toossi

**Affiliations:** 1 Translational Research Unit, Department of Experimental Research, Istituto Nazionale Malattie Infettive Lazzaro Spallanzani – IRCCS Rome, Italy; 2 Case Western Reserve University, Department of Medicine, Division of Infectious Diseases, Cleveland, Ohio, USA; 3 TBRU, Kampala, Uganda; 4 Clinical Epidemiology Unit, Department of Experimental Research, Istituto Nazionale Malattie Infettive Lazzaro Spallanzani – IRCCS Rome, Italy

## Abstract

**Background:**

Tuberculosis (TB) is the most frequent co-infection in HIV-infected individuals still presenting diagnostic difficulties particularly in developing countries. Recently an assay based on IFN-gamma response to *M. tuberculosis *RD1 peptides selected by computational analysis was developed whose presence is detected during active TB disease. Objective of this study was to investigate the response to selected RD1 peptides in HIV-1-infected subjects with or without active TB in a country endemic for TB and to evaluate the change of this response over time.

**Methods:**

30 HIV-infected individuals were prospectively enrolled, 20 with active TB and 10 without. Among those with TB, 12 were followed over time. IFN-gamma response to selected RD1 peptides was evaluated by enzyme-linked immunospot (ELISPOT) assay. As control, response to RD1 proteins was included. Results were correlated with immune, microbiological and virological data.

**Results:**

Among patients with active TB, 2/20 were excluded from the analysis, one due to cell artifacts and the other to unresponsiveness to *M. tuberculosis *antigens. Among those analyzable, response to selected RD1 peptides evaluated as spot-forming cells was significantly higher in subjects with active TB compared to those without (p = 0.02). Among the 12 TB patients studied over time a significant decrease (p =< 0.007) of IFN-gamma response was found at completion of therapy when all the sputum cultures for *M. tuberculosis *were negative. A ratio of RD1 peptides ELISPOT counts over CD4^+ ^T-cell counts greater than 0.21 yielded 100% sensitivity and 80% specificity for active TB. Conversely, response to RD1 intact proteins was not statistically different between subjects with or without TB at the time of recruitment; however a ratio of RD1 proteins ELISPOT counts over CD4^+ ^T-cell counts greater than 0.22 yielded 89% sensitivity and 70% specificity for active TB.

**Conclusion:**

In this pilot study the response to selected RD1 peptides is associated with TB disease in HIV-infected individuals in a high TB endemic country. This response decreases after successful therapy. The potential of the novel approach of relating ELISPOT spot-forming cell number and CD4^+ ^T-cell count may improve the possibility of diagnosing active TB and deserves further evaluation.

## Background

The World Health Organization has called for "urgent and extraordinary actions" to control tuberculosis (TB) in Africa [1]. Africa contains 9 of the 22 countries with the highest TB burden and the predominant factor driving the increased incidence of TB in these areas is the high prevalence of Human Immunodeficiency virus (HIV) infection [2-4].

HIV-1 co-infection significantly affects the progression of *M. tuberculosis *infection [5,6]. Innovative diagnostic tools for TB, new and enhanced treatment strategies, plus validation of markers that indicate efficacy of treatment, are needed to help combat the epidemic of dual HIV/TB co-infection. These need to be shown to be useful in TB-endemic settings.

A recent breakthrough in the diagnosis of *M. tuberculosis *infection has been the development of T-cell-based interferon(IFN)-gamma release assays (IGRAs) that use antigens belonging to *M. tuberculosis *region of difference-1 (RD1), including early secreted antigenic target-6 [ESAT-6] and culture filtrate protein 10 [CFP-10]). Two commercial IGRAs are now available, and evidence reviewed elsewhere [7-10] suggests that they are more specific than tuberculin skin test (TST), and correlate better with markers of TB infection in low incidence settings. Importantly, IGRAs are less affected by bacillus Calmette-Guerin (BCG) vaccination than the TST.

On the basis of this line of research, we recently reported an in vitro immune diagnostic enzyme-linked immunospot (ELISPOT) assay for IFN-gamma whose novelty consists in the use of RD1 peptides, which are multiepitopic and are selected by computational analysis [11-14]. The response to these peptides can be detected in subjects with ongoing *M. tuberculosis *replication, such as during active TB disease and/or recent infection, and decreases during TB therapy [15-17]. These studies conducted in Italy, a country with a low TB incidence (less than 10/100.000 population [18]), suggest that this assay may have a clinical value as a supplemental tool for diagnosis and monitoring of active TB. However, it is not known if this assay may be potentially useful also in a setting with high *M. tuberculosis *transmission. Moreover, it has been suggested that the clinical usefulness of assays measuring in vitro response to RD1 encoded antigens may be limited in patients with HIV-induced immunosuppression [19,20] although in studies in which ELISPOT-based assays were used, encouraging sensitivity (73–90%) for active HIV-associated TB was found in both children and adults [21-23].

Recently Rangaka et al. [24], reported an interesting approach to better identify patients with active TB among HIV^+ ^patients. This method directly correlates the ELISPOT results of RD1 proteins stimulation with the CD4^+ ^T-cell count of each single patient.

Thus, objectives of this pilot study in HIV-infected individuals from a tropical setting were: i) to evaluate whether this selected RD1 peptide assay may help in providing evidence of diagnosis of active TB in an endemic country; ii) to evaluate changes in the response to RD1 peptides during therapy; iii) to use the approach suggested by Rangaka et al. [24], as an additional tool for the identification of active TB; iv) to compare the results obtained with RD1 peptide assay with the response to RD1 intact proteins, an other in-house RD1 assay largely used in the literature [23-26].

## Methods

### Patient population and study design

HIV-1-infected adults with initial episode of sputum smear-positive pulmonary TB were recruited from the National Tuberculosis and Leprosy Programme Clinic at Mulago Hospital in Kampala, Uganda. Diagnosis of pulmonary TB was based on sputum culture confirmation. Pregnancy, extrapulmonary TB or chronic debilitating diseases, a Karnofsky performance scale score of ≤ 60 (patient unable to care for self and requiring the equivalent of hospital or institutional care), or CD4^+ ^T-cell counts of ≤ 100 cells/μl were exclusion criteria. Chest radiographs were interpreted by a single experienced chest physician, who was masked to the patients' clinical status and laboratory studies, using the standard scheme of the U.S. National Tuberculosis and Respiratory Disease Association and were classified as normal, minimal, moderately advanced, or far-advanced disease [27,28]. The study protocol was approved by the institutional review boards at University Hospitals of Cleveland Case Western Reserve University and the Uganda National AIDS Research Subcommittee. All subjects gave informed consent for study participation. Patients were treated with standard chemotherapy for pulmonary TB [27]. From January 2006 to January 2007, patients meeting the above-mentioned criteria were recruited.

TST was not performed in this study because of the poor specificity in areas of high BCG coverage like Uganda and where there is high prevalence of environmental mycobacteria.

No antiretroviral therapy was performed. Heparinized blood was obtained from subjects at enrolment and, in a smaller group (n = 12), at 6 months after initiation of treatment and in 9 of them also after 3 months of therapy. Complete blood counts and sputum examination were performed monthly during treatment. Follow-up chest radiographs were obtained at the end of the specific treatment. A group of healthy HIV-1-infected subjects without active TB (chest radiograph and sputum smear negative) with CD4^+ ^T-cell counts over 100 cells/μl were recruited as control group.

Plasma HIV-RNA load was detected by PCR by HIV Monitor test (1.5 Test) (Roche, Indianapolis, Indiana). CD4^+ ^T-cell counts were evaluated by immunostaining and fluorescence activated cell sorter (FACS) analysis [29].

### ELISPOT assays

The experimental procedures were conducted in Uganda at the Immunology laboratories of TB Reserarch Unit with fresh blood samples. The ELISPOT plates were shipped and read in Italy (see below). Briefly the peripheral blood mononuclear cells (PBMC) were isolated as described [15,16]. 2.5 × 10^5 ^PBMC were seeded (200 μL/well in duplicate) into microtiter 96 well UNIFILTER^® ^Microplates (Whatman plc, UK) pre-coated with capture antibody (Ab) (IFN-γ coating monoclonal, M-700A, Pierce-Endogen Inc. Rockford, Illinois). PBMC were then stimulated with ESAT-6 and CFP-10 peptide pools (50 μg/mL and 6 μg/mL respectively). ESAT-6 and CFP-10 intact proteins (2 μg/mL each) (Lionex, Germany) were used as positive controls for the specific responses to *M. tuberculosis *and were incubated overnight at 37°C, with 5% CO_2_. On the next morning, the cells were washed off, and the plates were incubated with a biotinylated capping antibody (Pierce-Endogen). On the next day, the plates were washed extensively and were incubated with streptavidin-horseradish peroxidase (Dako, Glostrup, Denmark). The plates then were developed with AEC substrate (Pierce), after a final washing step. Spots were then counted by an automated ELISA-Spot assay video analysis system (AELVIS, Hannover, Germany). Evaluated spots had a size >15 U (1 U = 50 μm^2^).

When the average number of spot-forming cells in duplicate wells of stimulated cultures exceeded that in controls by 10 spot-forming cells and, at the same time was above 34 spot-forming cells/million PBMC the responses were scored as positive [26]. To obtain the absolute value, the number of spot-forming cells in the negative controls was subtracted from the number of spot-forming cells in the stimulated cultures. Clinicians were blinded to the laboratory test results and laboratory personnel were blinded to the status of the patients.

### Statistical analysis

Median and range of values were calculated. The Mann-Whitney U test was used to compare continuous variables, and Chi square was used for categorical variables. Correlation between the response to the RD1 assays and CD4^+ ^T-cell counts was calculated by the Person's r coefficient. Analysis was carried out with SPSS v 14 for Windows (SPSS Italia srl, Bologna, Italy). Receiver-operator characteristic analysis was performed using Prism 4 software (GraphPad) [30].

## Results

### Characteristics of enrolled subjects

We enrolled 20 consecutive HIV-infected individuals with active pulmonary TB. Ten controls without active TB were also recruited. The demographic and clinical features of HIV-infected participants of the study are reported in Table 1. The two cohorts studied were not homogeneous in terms of CD4^+ ^T-cell counts (175/mm^3 ^vs. 393/mm^3^, active TB vs. controls without active TB, p = 0.001).

**Table 1:** Demographic and clinical characteristics of HIV-infected subjects enrolled in the study

	**Active TB** **N. 20 (%)**	**No Active TB** **N. 10 (%)**
**Female (%)**	13 (65)	8 (80)
**Age, median (years)**	32	31
**BCG vaccinated**	20 (100)	10 (100)
**HIV-RNA, median (cpmL)**	21675	11870
**CD4^+ ^T-cells**		
**Median (cells/μL)**	175	393
**101–200**	11 (55)	0
**201–300**	8 (40)	2 (20)
**≥ 301**	1 (5)	8 (80)

BCG: Bacillus Calmette and Guerin; HIV: Human immunodeficiency virus; Cpm: copies per ml; TB: tuberculosis.

### Response to selected RD1 peptides is associated with active TB

Among those with active TB, one sample was unreadable due to artifacts and one sample was deemed "unresponsive to *M. tuberculosis *antigens" (CD4^+ ^T-cell count: 154/mm^3^) because it tested negative to both selected RD1 peptides and intact proteins (used as positive control). Therefore these 2 samples were excluded from further analyses.

**Figure 1:**
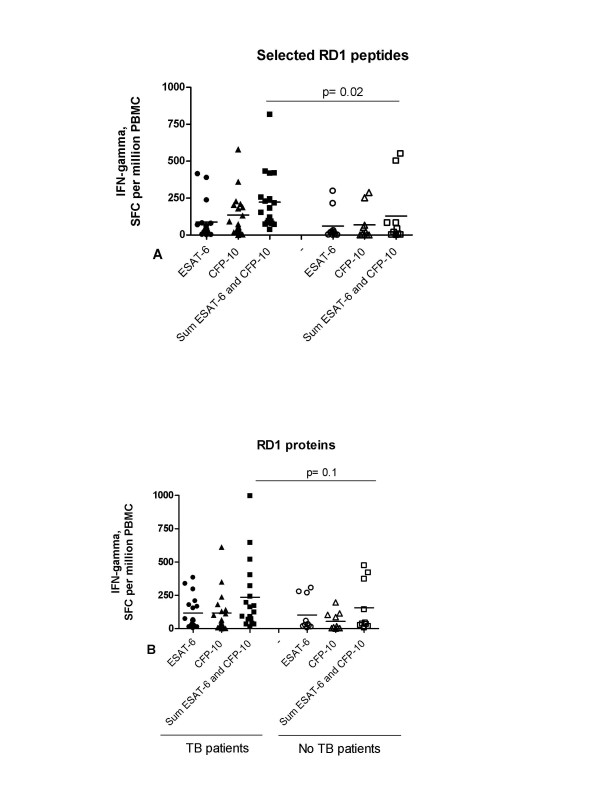
**A-B IFN-gamma ELISPOT response to antigens of *Mycobacterium tuberculosis *in HIV^+ ^subjects**. **A) **Response to selected RD1 peptides and **B) **to RD1 proteins, are shown as spot-forming cells (SFC) per 10^6 ^PBMCs. Shaded symbols indicate individuals with tuberculosis (TB patients) and unshaded symbols indicate individuals without tuberculosis (No active TB). Horizontal lines indicate median values. The TB patient group had a significantly higher response to the sum of ESAT-6 and CFP-10 peptides compared with the group without active TB (p = 0.02).

Among the 28 subjects analyzed, 18 with active TB and 10 without active TB, the median responses to selected ESAT-6 peptides (40 spot-forming cells [interquartile range {IQR}, 0–414 spot-forming cells] vs. 17 spot-forming cells [IQR, 0–298 spot-forming cells] p = 0.09) and selected CFP-10 peptides (83 spot-forming cells [IQR, 18–580 spot-forming cells] vs. 14 spot-forming cells [IQR, 0–288 spot-forming cells]; p = 0.1) were not significantly higher in the group with active TB, compared with the control group. Conversely the sum of ESAT-6 and CFP-10 peptide responses (167 spot-forming cells [IQR, 38–816 spot-forming cells] vs. 30 spot-forming cells [IQR, 10–550 spot-forming cells]; p = 0.02) was significantly higher in the group with active TB, compared with the group without active disease (Figure 1A).

As internal control, the response to RD1 proteins was evaluated. The median responses to ESAT-6 protein (63 spot-forming cells [interquartile range {IQR}, 4–384 spot-forming cells] vs. 31 spot-forming cells [IQR, 6–306 spot-forming cells] p = 0.5), CFP-10 protein (63 spot-forming cells [IQR, 0–612 spot-forming cells] vs. 14 spot-forming cells [IQR, 0–196 spot-forming cells]; p = 0.1) and the sum of ESAT-6 and CFP-10 protein responses (143 spot-forming cells [interquartile range {IQR}, 14–996 spot-forming cells] vs. 40 spot-forming cells [IQR, 6–474 spot-forming cells] p = 0.2), were not significantly different in the two groups, with or without active TB (Figure 1B).

Next we evaluated whether there was a correlation between the response to the selected RD1 peptides and the virological status of the patients. An inverse correlation between the response to RD1 peptides and HIV load was found although not statistically significant (p = 0.06).

Thus, based on these results, the response to the combined ESAT-6 and CFP-10 peptides was associated with active TB better than the response to the single pool of peptides and proteins (either considered singularly or as the response to the sum of ESAT-6 and CFP10 proteins). Consequently, hereafter, we report only the analysis of the sum of ESAT-6 and CFP-10 data, either peptides or proteins.

### Evaluation of the response to RD1 epitopes over time and correlation with clinical, microbiological and radiological data

To evaluate changes in T-cell responsiveness to RD1 peptides during TB treatment 12 cases were followed over time, at the time of TB diagnosis and after 6 months of successful therapy leading to culture sputum negativization. Nine of them were studied also after 3 months of therapy.

**Figure 2:**
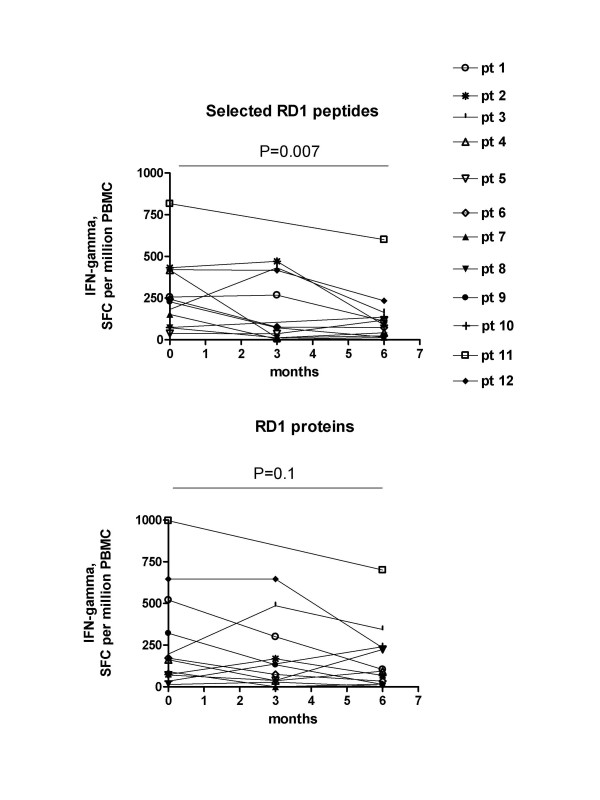
**A-B. Significant decrease of IFN-gamma response to selected RD1 peptides over time in HIV^+ ^patients with active TB disease**. **A) **Response to the sum of ESAT-6 and CFP-10 peptides and **B) **to the sum of ESAT-6 and CFP-10 proteins in terms of IFN-gamma spot-forming cells (SFCs) per million PBMC for each individual at the time of diagnosis and 6 months after therapy are reported. For 9/12 individuals data are reported also after 3 months of therapy. The p value denotes the difference between the responders in each group.

The median of the sum of ESAT-6 and CFP-10 peptide response at diagnosis (236 [IQR, 38–816 spot-forming cells], after 3 months of therapy (74 [IQR, 4–470 spot-forming cells]) and after 6 month at therapy completion (100 [IQR, 14–602 spot-forming cells] were analyzed (Figure 2A). A significant decrease of the response was found between baseline and 6 months after therapy (p = 0.007). If the responses were evaluated by a qualitative scoring, a conversion to negative at the completion of treatment was found in 3/12 patients.

When the response to RD1 proteins was evaluated, no statistically significant change was observed over time, with median values of 167 [IQR, 14–996 spot-forming cells], 103 [IQR, 0–646 spot-forming cells] and 100 [IQR, 2–698 spot-forming cells] respectively at diagnosis and after 3 and 6 months of therapy (Figure 2B) (p = 0.1 for the comparison of the results at the time of diagnosis vs. completion of therapy). If the responses were evaluated by a qualitative scoring, a conversion to negative at the completion of treatment was found in 3/12 patients.

Next we evaluated the relationship between the RD1 responses and microbiological and radiological features. No correlation was found between the quantitative response to selected RD1 peptides, *M. tuberculosis *load in sputum (smear and culture) and the radiological lesions (evaluated by the extension of lung lesions [28]) (data not shown).

Based on the results described, we conclude that a successful therapy for TB causes a significant decrease of the sum of ESAT-6 and CFP-10 selected peptide response.

#### Stratification of responses by CD4^+ ^T-cell counts

The response to RD1 tests are mainly CD4-mediated [16], and will therefore be influenced by the absolute CD4^+ ^T-cell count, especially in those with HIV infection. We therefore stratified the ELISPOT results by CD4^+ ^T-cell counts.

**Figure 3:**
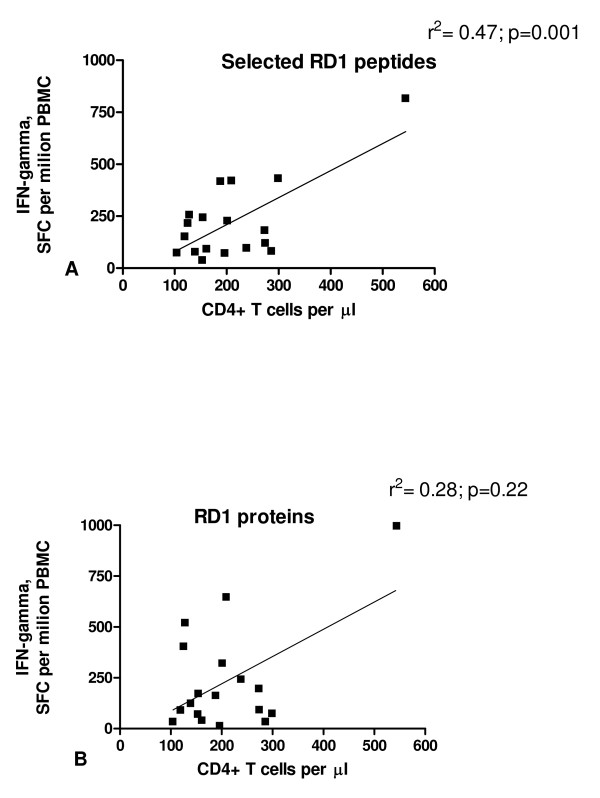
**A-B. Significant correlation between the response to RD1 antigens and CD4^+ ^T-cell count in HIV-infected individuals with active-TB**. **A) **Response to the sum of ESAT-6 and CFP-10 peptides and **B) **to the sum of ESAT-6 and CFP-10 proteins in terms of IFN-gamma spot-forming cells (SFCs) per million PBMC for each individual are reported and correlated with the CD4^+ ^T-cell count per mm^3^. The p value denotes the statistical significance of the correlation between CD4^+ ^T-cell count and the response to the assay.

Among subjects with active TB, the CD4^+ ^T-cell count significantly correlated with the sum of ESAT-6 and CFP-10 peptide response (r^2^:0.47; p = 0.001) (Figure 3A), and with the sum of ESAT-6 and CFP-10 protein responses (r^2^:0.28; p = 0.02) (Figure 3B). However no firm conclusions can be drawn on the correlation between the CD4+ T-cell counts and RD1 responses, as the exclusion of one outlier result (one subject with very high CD4+ T-cell counts and spot-forming cells for peptides and proteins, 544/mm^3^, 816 and 996 spot-forming cells per million PBMC respectively) renders the correlation coefficients not statistically significant for both responses to selected RD1 peptides (r^2^:0.04; p = 0.4) and proteins (r^2^:0.01; p = 0.6).

**Figure 4:**
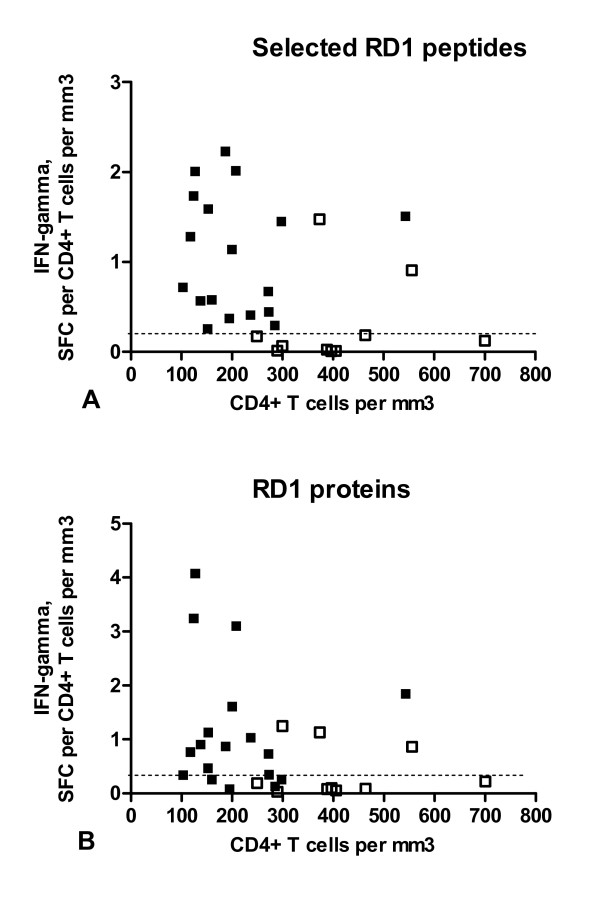
**A-B. A higher ratio of the response to RD1 antigens in relation to CD4^+ ^T-cell count is associated with active TB in HIV^+ ^subjects**. ELISPOT counts per million PBMC for each antigen were divided by the corresponding total CD4^+ ^T-cell count per mm^3 ^and were plotted against CD4^+ ^T-cell counts. Shaded symbols indicate patients with TB and unshaded symbols indicate individuals without TB. **A) **For the response to selected RD1 peptides, a ratio in the TB group (shaded symbols) higher than 0.21 predicted active TB with 100% sensitivity and 80% specificity). **B) **For the response to RD1 proteins, a ratio in the TB group (shaded symbols) higher than 0.22 predicted active TB with 89% sensitivity and 70% specificity.

**Figure 5:**
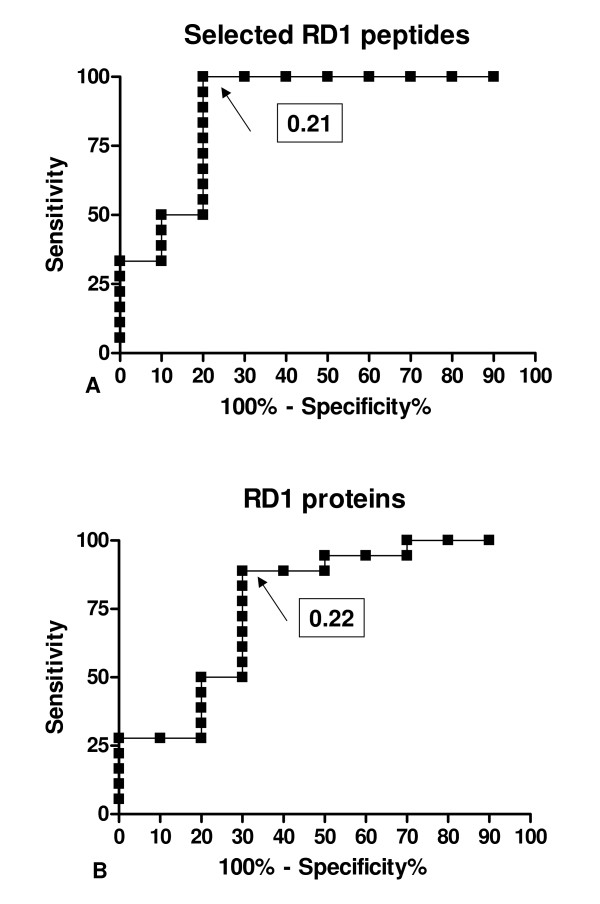
**A-B. ROC curves of the tests based on the ratio of the response to RD1 antigens in relation to CD4^+ ^T-cell counts**. A ratio defined by the result obtained when the ELISPOT counts per million PBMC for each antigen were divided by the corresponding total CD4^+ ^T-cell count per mm^3 ^was used to perform a receiver-operator characteristic analysis, using the group with active TB and the subjects without active disease as comparator groups. **A) **ROC curve for the response to RD1 selected peptides. A ratio higher than 0.21 predicted active TB with 100% sensitivity and 80% specificity. **B) **ROC curve for the response to RD1 intact proteins. A ratio higher than 0.22 predicted active TB with 89% sensitivity and 70% specificity.

Considering the approach suggested by Rangaka et al. [24], we then calculated the frequency of the RD1 responses in relationship to the CD4^+ ^T-cell counts. In particular the ratio defined by the ELISPOT counts (per million PBMC) for each antigen divided by the CD4^+ ^T-cell counts was determined. The median value of these ratios for both assays, RD1 peptides and proteins, was significantly higher in the active TB group than that in the control group (Table 2). Then we explored the possibility of using this approach to better identify the patients with active TB. We used the ratio data to perform a receiver-operator characteristic (ROC) analysis, using the groups with and without active disease as comparator groups (Figure 4A–B). Significant results for area under the curve (AUC) analysis were obtained for the sum of ESAT-6 and CFP-10 peptides (AUC, 0.88; P = 0.0009; 95% CI, 0.72–1.0). Interestingly, a ratio higher than 0.21 predicted active TB with 100% sensitivity (95% CI, 81%–100%) and 80% specificity (95% CI, 44%–97%) (Figure 5A).

**Table 2:** IFN-gamma ELISPOT ratio between the sum of ESAT-6 and CFP-10 peptides SFC per million PBMC and the CD4^+ ^T-cell counts/mm^3 ^in patients with or without active TB.

	**Median of IFN-gamma ELISPOT ratio (interquartile range)**^a^		
	**Active TB** **N = 18**	**No Active TB** **N = 10**	**p**
**Sum of ESAT-6 and CFP-10 peptides**	0.92 (0.24–2.23)	0.08 (0–1.46)	0.001^b^
**Sum of ESAT-6 and CFP-10 proteins**	0.80 (0.07–4)	0.13 (0.02–1.23)	0.02^b^

^a ^IFN-gamma ELISPOT ratio (spot-forming cells per 10^6 ^PBMCs) for each antigen was divided by the total CD4^+ ^T-cell count per mm^3^, as previously shown (24). ^b ^Statistically significant.

TB: tuberculosis.

In addition when considering the sum of ESAT-6 and CFP-10 proteins, (AUC, 0.77; P = 0.01; 95% CI, 0.57–0.97), a ratio higher than 0.22 predicted active TB with 89% sensitivity (95% CI, 81%–100%) and 70% specificity (95% CI, 34%–93%) (Figure 5B). No statistically significant difference was found between the AUC of the RD1 peptides and protein assay (p = 0.4), although the accuracy of the RD1 peptide test was higher.

Thus, based on these results, in HIV^+ ^patients the use of a ratio of combined ESAT-6 and CFP-10 ELISPOT count divided by the CD4^+ ^T-cell count may be a more accurate tool for the diagnosis of active TB compared to the use of the number of spot-forming cells. Moreover, by this approach the accuracy of the assay based on RD1 peptides is higher compared to that based on RD1 proteins.

## Discussion

This pilot study demonstrates that also in developing countries the response to selected RD1 peptides is associated with active pulmonary TB in HIV-infected individuals. Moreover the response to peptides significantly decreases after efficacious therapy for TB disease. Furthermore a ratio of combined ESAT-6 and CFP-10 peptide ELISPOT count divided by the CD4^+ ^T-cell count greater than 0.21 had 100% sensitivity and 80% specificity for active pulmonary TB. To our knowledge this is the first study performed in a TB endemic region of the world in which a concomitant evaluation of the response to a test based on RD1 antigens, CD4^+ ^T-cell counts, HIV load and performance over time of sputum culture for *M. tuberculosis *was carried out in HIV^+ ^patients with active TB.

In this study the RD1 response correlates with the CD4^+ ^T-cell number, although weakly. The patients studied had CD4^+ ^T-cell counts above 100/μl. Among them the HIV-related immunosuppression did not affect the qualitative response to selected RD1 peptides. In fact among those with active TB only one patient resulted incapable of responding to *M. tuberculosis*-specific antigens. Moreover the absolute number of spot-forming cells in response to the RD1 peptide was significantly lower in those without active TB compared to those with active disease in spite of a significant higher number of CD4^+ ^T-cells. This indicates that the test is not influenced by CD4^+ ^T-cell counts and has good chances of identifying active TB. Nevertheless, an impairment of this assay may be expected in presence of severe immunosuppression (i.e. CD4^+ ^T-cell counts under 100/μl), as previously reported [19,31].

From the literature it is known that commercial RD1 assays, based on intact CFP-10 and ESAT-6 proteins and on pools of overlapping peptides spanning the whole length of these proteins [23-26], detect both latent TB infection (LTBI) and active TB disease [20-26]. Conversely the response to the selected RD1 peptides is associated with active *M. tuberculosis *replication [15-17]. These differences may be related to the amount and the composition of epitopes covered by the different peptides or by the whole intact protein used in the diverse tests. As previously reported the response to the selected RD1 peptides is mediated by CD4^+ ^T effector cells, shown to undergo clonal expansion during *M. tuberculosis *replication, followed by a contraction phase after efficacious therapy culminating in the generation of CD4^+ ^memory T-cells [16]. The present study shows for the first time that RD1 T-cell responses decrease in HIV/TB patients after successful therapy for TB, confirming the results obtained in patients without HIV disease [32-35]. The trend of decrease is in line with previous reports using the ELISPOT technology [32-35] indicating that these responses better correlate with bacterial burden than whole blood tests [36]. Here conversion to negative response to selected RD1 peptides was found only in 30% of the patients analyzed, differently from our previous study in which this was found in 100% of the patients after successful therapy [15]. This may relate to different settings under which the test was done, as reported by others [36]. In particular a potential re-exposure to *M. tuberculosis*, a likely scenario in endemic countries, after the completion of treatment or to environmental mycobacteria, commonly encountered in the tropics, which share the esat-6 and cfp-10 genes may account for the diverse finding.

A decrease of RD1-specific CD4^+ ^T-cell effector responses has been reported also in animal models of *M. tuberculosis *infection in which a decrease in responses in mononuclear cells from both the lymph nodes and lungs after the acute phase of infection was shown [37]. This model has been confirmed by studies performed in patients with diseases different from TB such as HIV-infected patients undergoing structured treatment interruption [38], in whom HIV-specific T-cell responses have been reported to be higher at the peak of viremia and then to decrease when therapy is reintroduced [39]. Similarly, in patients who had been acutely infected with Hepatitis C Virus and who cleared the infection, high frequencies of effector T-cells are found soon after infection, whereas higher frequencies of memory T-cells appear later on [40].

According to the literature [24], our analysis of these responses suggests that the ELISPOT test could be incorporated into practice in the context of HIV infection. A positive result to the sum of ESAT-6 and CFP-10 peptide response per 10^6 ^PBMC divided by the CD4^+ ^T-cell count greater than 0.21 would strongly suggest active disease that should be investigated and treated. In the present study, the ratio found for the assay based on intact RD1 proteins is different compared to that reported by Rangaka et al. [24], probably reflecting the fact that the assay based on RD1 proteins is not standardized (different concentration of proteins and ELISPOT reagents). However since the response to RD1 tests are mainly CD4-mediated [16], we think it is important, especially in HIV^+ ^patients, to analyze the RD1 responses in a context of CD4^+ ^T-cell number, at least for those tests that are still not standardized [24,41].

Specificity of the test based on selected RD1 peptides was 80%. False positive results may be attributed at least in part to the high prevalence of LTBI and/or elevated probability of high risk exposure to *M. tuberculosis *that can be expected in high TB endemic countries. It would be important to evaluate if this assay is capable of predicting onset of TB disease in those without active TB that resulted positive. Currently all the controls of this study are followed over time.

This is a pilot study which presents limitations that will require additional work to be overcome. Above all, the study was performed on a relatively small number of individuals and certainly the research would benefit from a larger study taking into account type of antiretroviral therapy, if performed, and concomitant diseases. Nevertheless this pilot study comprised several tests with several correlations performed (two RD1 tests with multiple antigens; correlation with CD4^+ ^T-cell count; comparison with virological and microbiological data) indicating that the response to selected RD1 peptide assay is associated with active pulmonary TB in HIV-infected individuals from an African country.

## Conclusion

In conclusion the assay based on selected RD1 peptides may be useful in the design of larger studies in the tropical setting, to better evaluate the performance of such test in countries at high endemia of TB.

## List of abbreviations

• AUC: area under the curve

• BCG: Bacillus Calmette Guerin

• Cpm: copies per ml

• CI: confidence interval

• CFP: culture filtrate protein

• ELISPOT: enzyme linked immune spot

• ESAT: early secreted antigenic target

• FACS: fluorescence activated cell sorter

• HIV: Human Immunodeficiency Virus infection

• IFN: interferon

• IGRA: IFN-gamma release assays

• IQR: interquartile range

• LTBI: Latent tuberculosis infection

• PBMC: peripheral blood mononuclear cells

• RD: region of difference

• RD1: CFP-10 and ESAT-6

• ROC: receiver-operator characteristic

• SFC: spot-forming cells

• TST: tuberculin skin test

• TB: Tuberculosis

## Competing interests

DG, SC and EG have a patent pending on T-cell assay based on selected RD1 peptides.

## Authors' contributions

DG designed the study, performed data analysis and wrote the draft of the manuscript, SC helped in performing data analysis and writing the draft of the manuscript, MM carried out the data base of collected data and helped in the statistical analysis, JB carried out the immunological assays, HMK helped in the recruitment of subjects and administration of studies oversees, EG helped in performing the statistical analysis and writing the draft of the manuscript, ZT conceived of the study, designed the study, supervised the experimental work and helped to draft the manuscript. The article has not been submitted elsewhere and all co-authors have read and approved the final manuscript with its conclusions.

## Pre-publication history

The pre-publication history for this paper can be accessed here:


